# Supplementary material for: DTX3L-mediated TIRR nuclear export and degradation regulates DNA repair pathway choice and PARP inhibitor sensitivity
Source: Nat Commun. 2024 Dec 5;15:10596. doi: 10.1038/s41467-024-54978-5 (PMC11618752; doi:10.1038/s41467-024-54978-5)
Supplement: Supplementary file 1 — Supplementary Information [file 41467_2024_54978_MOESM1_ESM.pdf]

Supplementary Materials for

DTX3L-mediated TIRR nuclear export and degradation regulates DNA repair pathway choice and PARP inhibitor sensitivity

Qi Ye, Jian Ma, Zixi Wang, Lei Li, Tianjie Liu, Bin Wang, Lizhe Zhu, Yuzeshi Lei, Shan Xu, Ke Wang, Yanlin Jian, Bohan Ma, Yizeng Fan, Jing Liu, Yang Gao, Haojie Huang, Lei Li

Correspondence to: Jian Ma (majian0922@gmail.com); Haojie Huang (huanghaojie@zju.edu.cn); Lei Li (lilydr@163.com)

This PDF file includes:

Supplementary Figure 1 to 6
Supplementary Table 1

Supplementary Figure 1

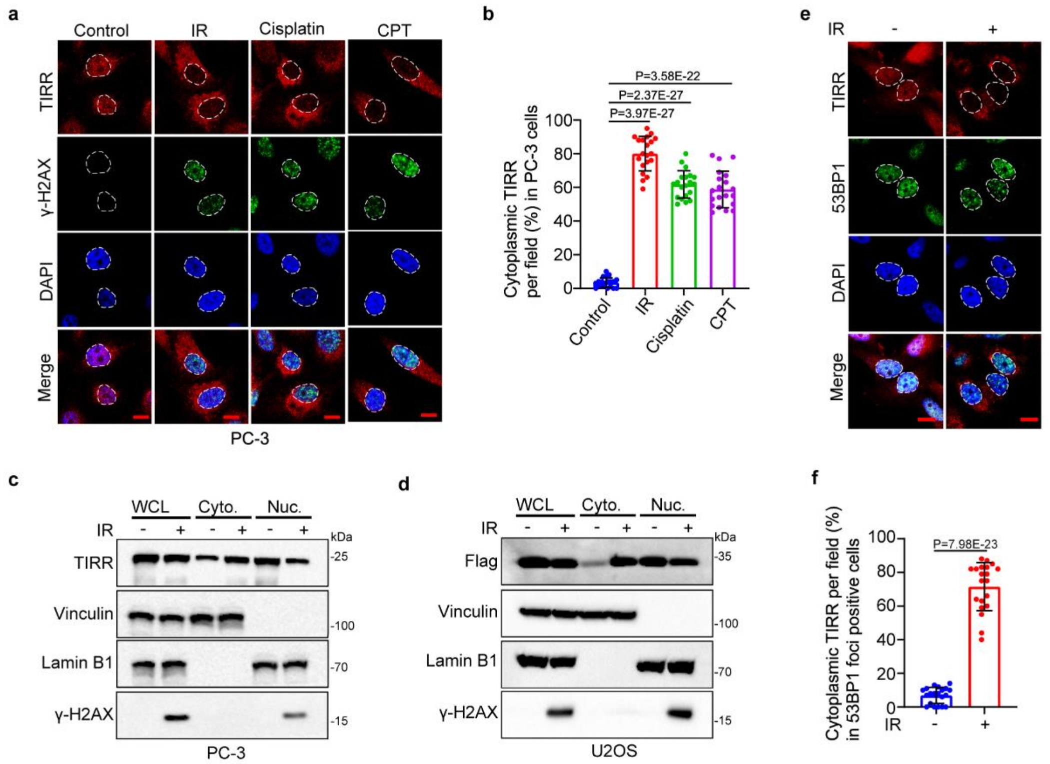

Supplementary Fig. 1 TIRR is translocated to the cytoplasm after DNA damage. Related to Fig 1.

a IFC of TIRR and γ-H2AX in PC-3 cells treated with IR (8 Gy), Cisplatin (10 μM, 24 hours) or CPT (2 μM, 3 hours). Scale bar, 10 μm.

b Quantification of cells with cytoplasmic translocation of endogenous TIRR as shown in (**a**). Data were shown as the mean ± SD from 20 fields (>200 cells, n = 20) from three biological replicates. Two-tailed unpaired Student's *t*-test; *P* values based on the order of appearance: 3.97E-27, 2.37E-27, 3.58E-22.

c WB analysis of whole-cell (WCL), cytosolic (Cyto.) and nuclear (Nuc.) fractions from PC-3 cells with or without IR (8 Gy).

d WB analysis of whole-cell (WCL), cytosolic (Cyto.) and nuclear (Nuc.) fractions from U2OS cells transfected with Flag-TIRR, and treated with or without IR (8 Gy).

e IFC of TIRR and 53BP1 in PC-3 cells treated with IR (8 Gy). Scale bar, 10 μm.

f Quantification of cells with positive 53BP1 foci and endogenous TIRR cytoplasmic translocation (**e**). Data were shown as the mean ± SD from 20 fields (>200 cells, n = 20) from three biological replicates. Two-tailed unpaired Student's *t*-test; *P* = 7.98E-23. Source data are provided as a Source Data file. Similar results for (**c, d**) panels were obtained in three independent experiments.

Supplementary Figure 2

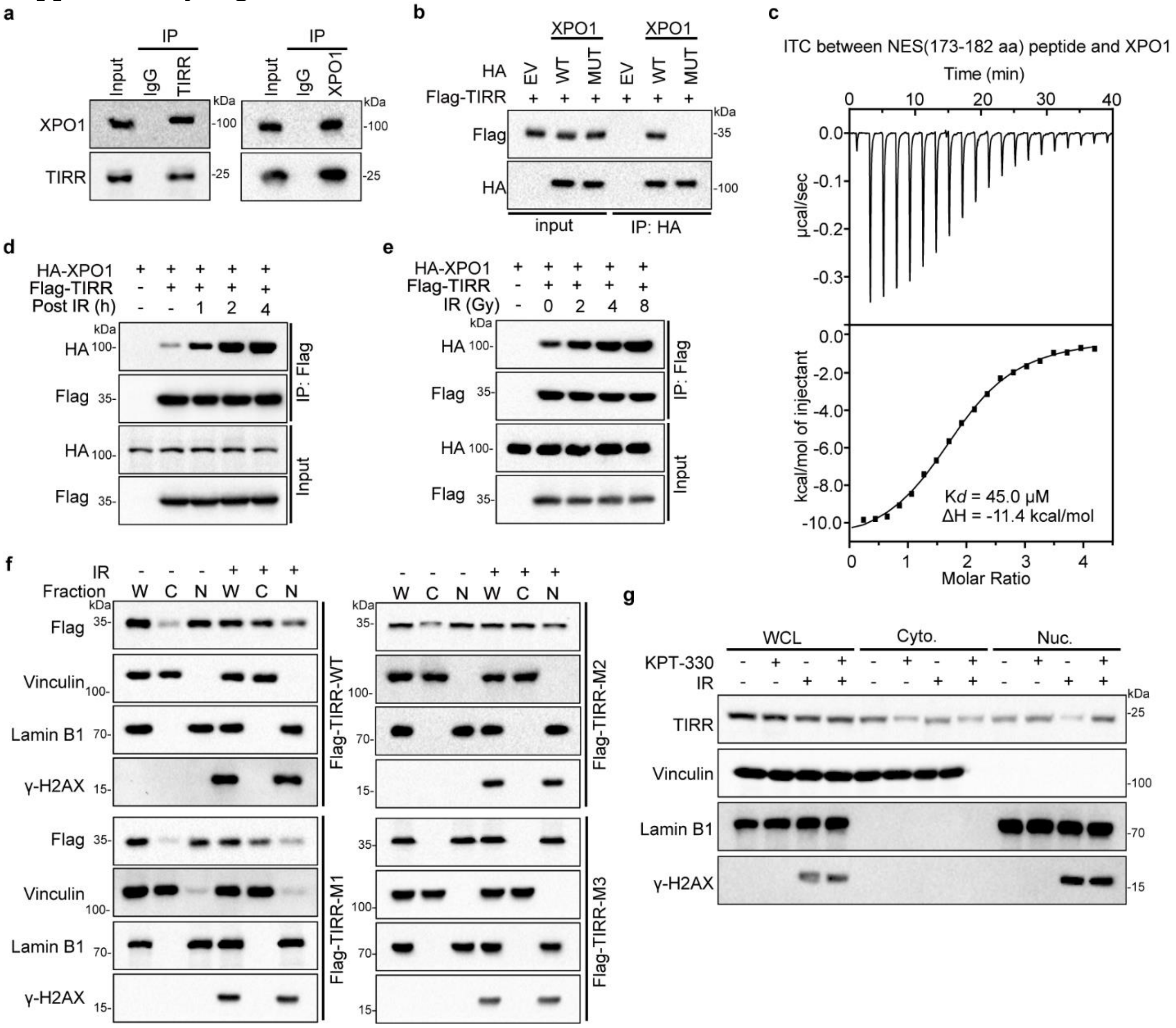

Supplementary Fig. 2 XPO1 mediates TIRR nuclear exporting in response to DNA damage. Related to Fig 2.

a Co-IP of endogenous TIRR and XPO1 in whole-cell lysates from PC-3 cells.

b Co-IP of whole-cell lysates from 293T cells transfected with Flag-TIRR, HA-XPO1-WT or HA-XPO1 mutant (I521A/L525A/F561A/F572A).

c Binding affinity measured by isothermal titration calorimetry (ITC) between XPO1 and the synthesized NES peptide (173-182 aa of TIRR).

d Co-IP of whole-cell lysates from 293T cells transfected with Flag-TIRR and HA-XPO1, and harvested at indicated time points post-IR (8 Gy).

e Co-IP of whole-cell lysates from 293T cells transfected with Flag-TIRR and HA-XPO1, treated with varying doses of IR from 0 to 8 Gy.

f WB analysis of whole-cell (W), cytosolic (C) and nuclear (N) fractions from 293T cells transfected with Flag-TIRR-WT or its NES mutants, followed by IR (8 Gy).

g WB analysis of whole-cell (WCL), cytosolic (Cyto.) and nuclear (Nuc.) fractions from PC-3 cells pretreated with or without KPT-330 (1 μM, 12 hours), followed by IR (8 Gy). Source data

are provided as a Source Data file. Similar results for (**a, b, d-g**) panels were obtained in three independent experiments.

Supplementary Figure 3

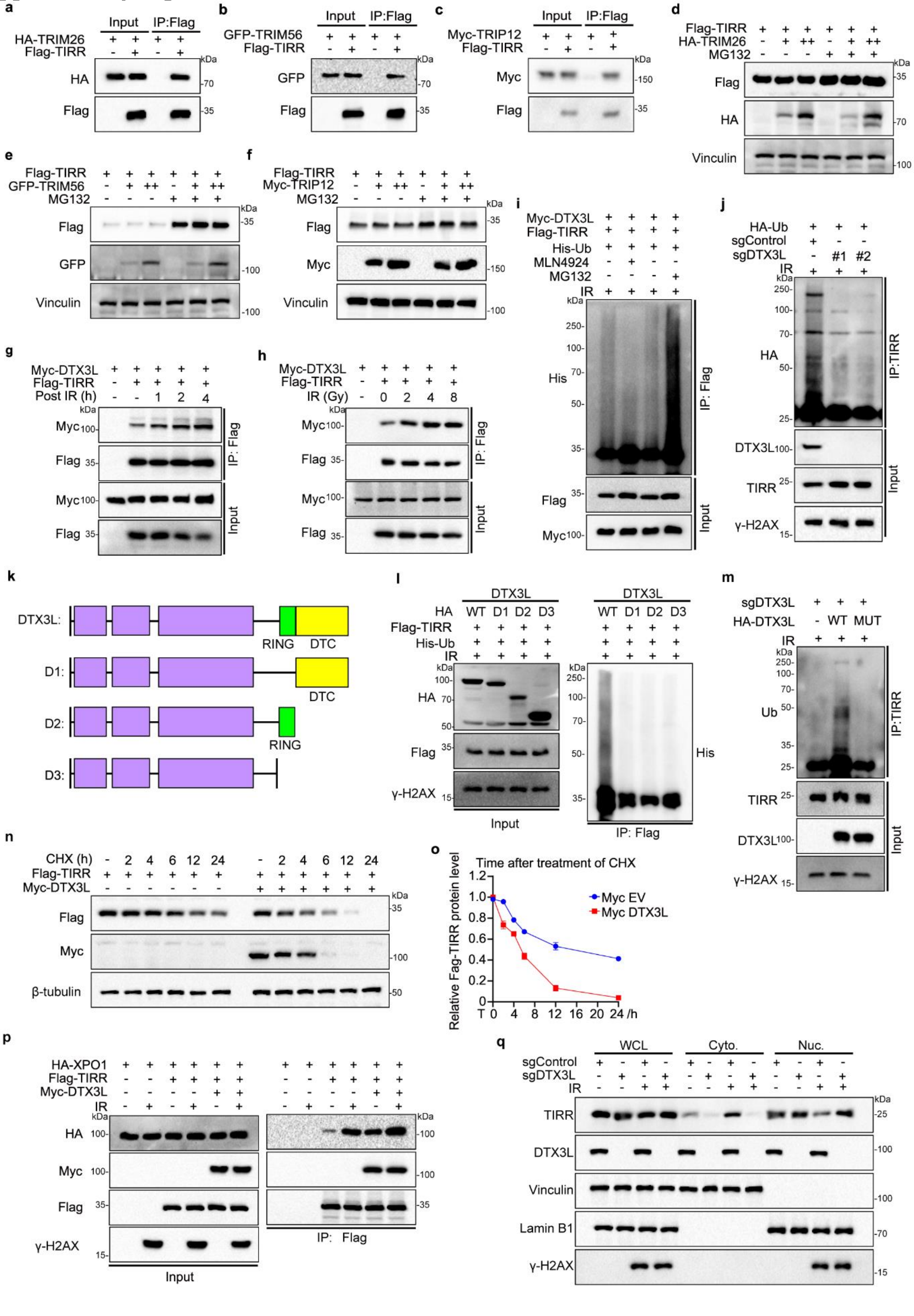

Supplementary Fig. 3 DTX3L-mediated TIRR ubiquitination triggers TIRR nuclear translocation and degradation after DNA damage. Related to Fig. 4.

a-c Co-IP of whole-cell lysates from 293T cells transfected with Flag-TIRR, HA-TRIM26, GFP-TRIM56 or Myc-TRIP12.

d-f WB analysis of whole-cell lysates from 293T cells transfected with Flag-TIRR, HA-TRIM26, GFP-TRIM56 or Myc-TRIP12, pretreated with or without MG132, and harvested 2 hours post-IR (8 Gy).

g Co-IP of whole-cell lysates from 293T cells transfected with Flag-TIRR and Myc-DTX3L at indicated time points post-IR (8 Gy).

h Co-IP of whole-cell lysates from 293T cells transfected with Flag-TIRR and Myc-DTX3L, treated with varying doses of IR (0 ~ 8 Gy).

i In vivo polyubiquitination assay of whole-cell lysates from 293T cells transfected with HA-Ub, Myc-DTX3L, Flag-TIRR, pretreated with DMSO, MG132 or MLN4924, and harvested after IR (8 Gy).

j In vivo polyubiquitination assay of whole-cell lysates from control or DTX3L knockout PC-3 cells transfected with HA-Ub, pretreated with MG132 and exposed to IR (4 Gy).

k Diagram showing wild-type DTX3L and mutant constructs with deletions in the RING (green) or DTC (yellow) domain.

l In vivo polyubiquitination assay of whole-cell lysates from 293T cells transfected with Flag-TIRR, His-Ub, HA-DTX3L WT or D1, D2, D3 mutant, pretreated with MG132 and exposed to IR (8 Gy).

m In vivo polyubiquitination assay of whole-cell lysates from DTX3L depleted PC-3 cells transfected with HA-DTX3L WT or catalytic inactive mutant (C561S/C564S), pretreated with MG132 and exposed to IR (8 Gy).

n, o WB analysis (**n**) of whole-cell Flag-TIRR protein after CHX treatment and quantification (**o**) in 293T cells transfected with Flag-TIRR and Myc-DTX3L. Data were shown as the mean ± SD from three biological replicates (n = 3).

p Co-IP of whole-cell lysates from 293T cells transfected with Flag-TIRR, Myc-DTX3L and HA-XPO1, with or without IR (8 Gy).

q WB analysis of whole-cell (WCL), cytosolic (Cyto.) and nuclear (Nuc.) fractions from control or DTX3L knockout PC-3 cells after IR (8 Gy). Source data are provided as a Source Data file. Similar results for (**a-j, l-n, p, q**) panels were obtained in three independent experiments.

Supplementary Figure 4

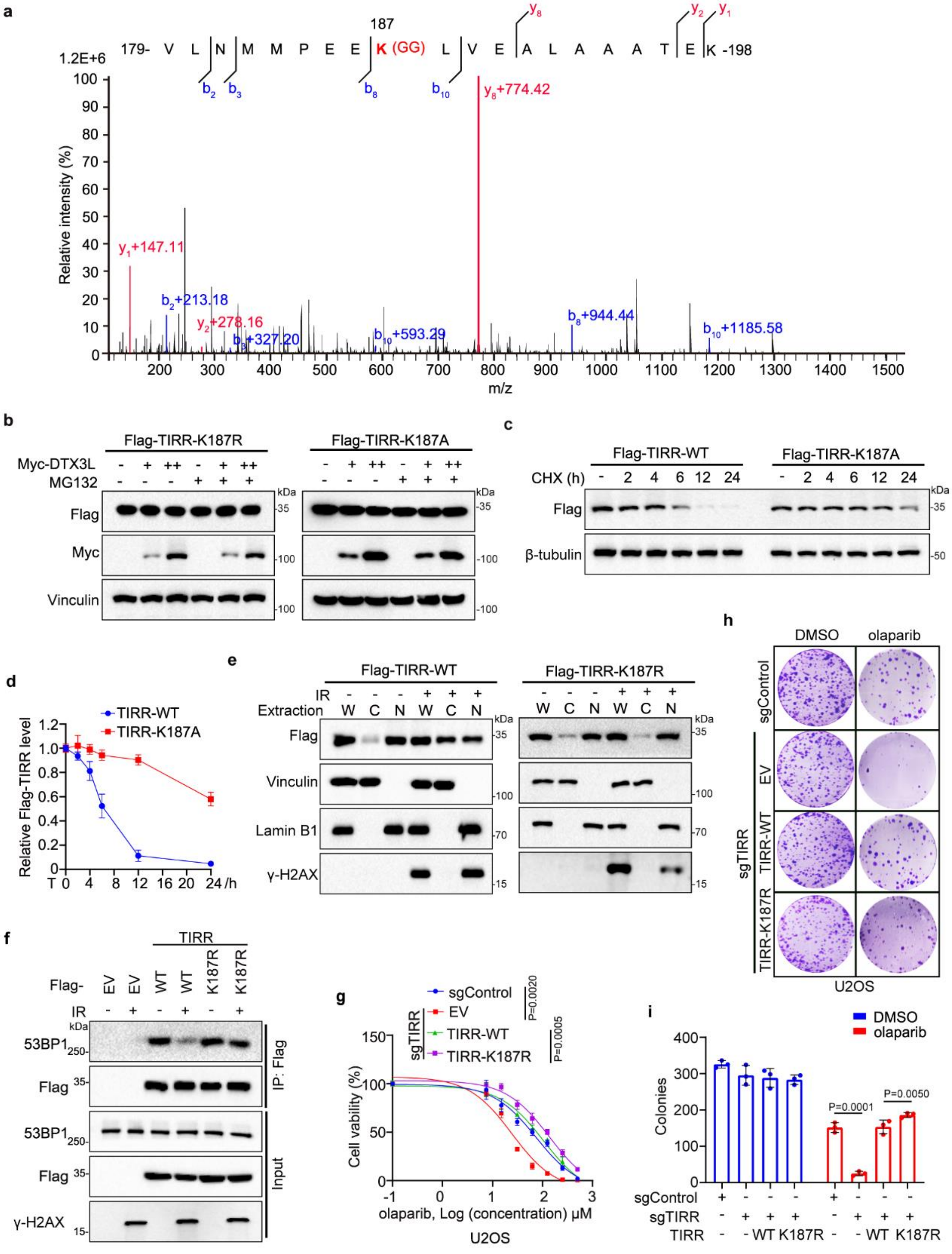

Supplementary Fig. 4 Ubiquitination of TIRR at lysine-187 by DTX3L dictates NHEJ pathway activity and PARP inhibitor sensitivity. Related to Fig. 5.

a Representative annotated LC-MS/MS spectra of TIRR peptide 179-VLNMMPEEKLVEALAAATEK-198, containing a Gly-Gly modified (ubiquitinated) lysine-187.

b WB analysis of Flag-TIRR in whole-cell lysates from 293T cells transfected with Flag-TIRR K187R or K187A mutant, and pretreated with or without MG132 treatment, 2 hours post-IR (8 Gy).
c, d WB analysis of whole-cell Flag-TIRR WT or K187A mutant protein from 293T cells at indicated time points of CHX treatment (**c**), with corresponding quantification analysis (**d**). Data were shown as the mean ± SD from three biological replicates (n = 3).
e WB analysis of whole-cell (W), cytosolic (C) and nuclear (N) fractions from 293T cells transfected with Flag-TIRR-WT or K187R mutant, and treated with or without IR (8 Gy).
f Co-IP of whole-cell lysates from 293T cells transfected with Flag-TIRR-WT or K187R mutant, and treated with or without IR (8 Gy).
g Dose-response survival curves of control or TIRR knockout U2OS cells transfected with EV, Flag-TIRR-WT or K187R mutant exposed to increasing concentrations of olaparib. Data were shown as the mean ± SD of three independent experiments (n = 3). Two-way ANOVA; *P* values based on the order of appearance, *P* = 0.0020, F = 51.98; *P* = 0.0005, F =108.8.
h, i Colony formation assays in control or TIRR knockout U2OS cells transfected with EV, Flag-TIRR-WT or K187R mutant. The number of colonies was counted. Representative colonies are shown in (**h**), with quantification data shown in (**i**). Data were presented as the mean ± SD of three independent experiments (n = 3). Two-tailed unpaired Student's *t*-test. *P* values based on the order of appearance: 0.0001,0.0050. Source data are provided as a Source Data file. Similar results for (**b, c, e, f**) panels were obtained in three independent experiments.

Supplementary Figure 5

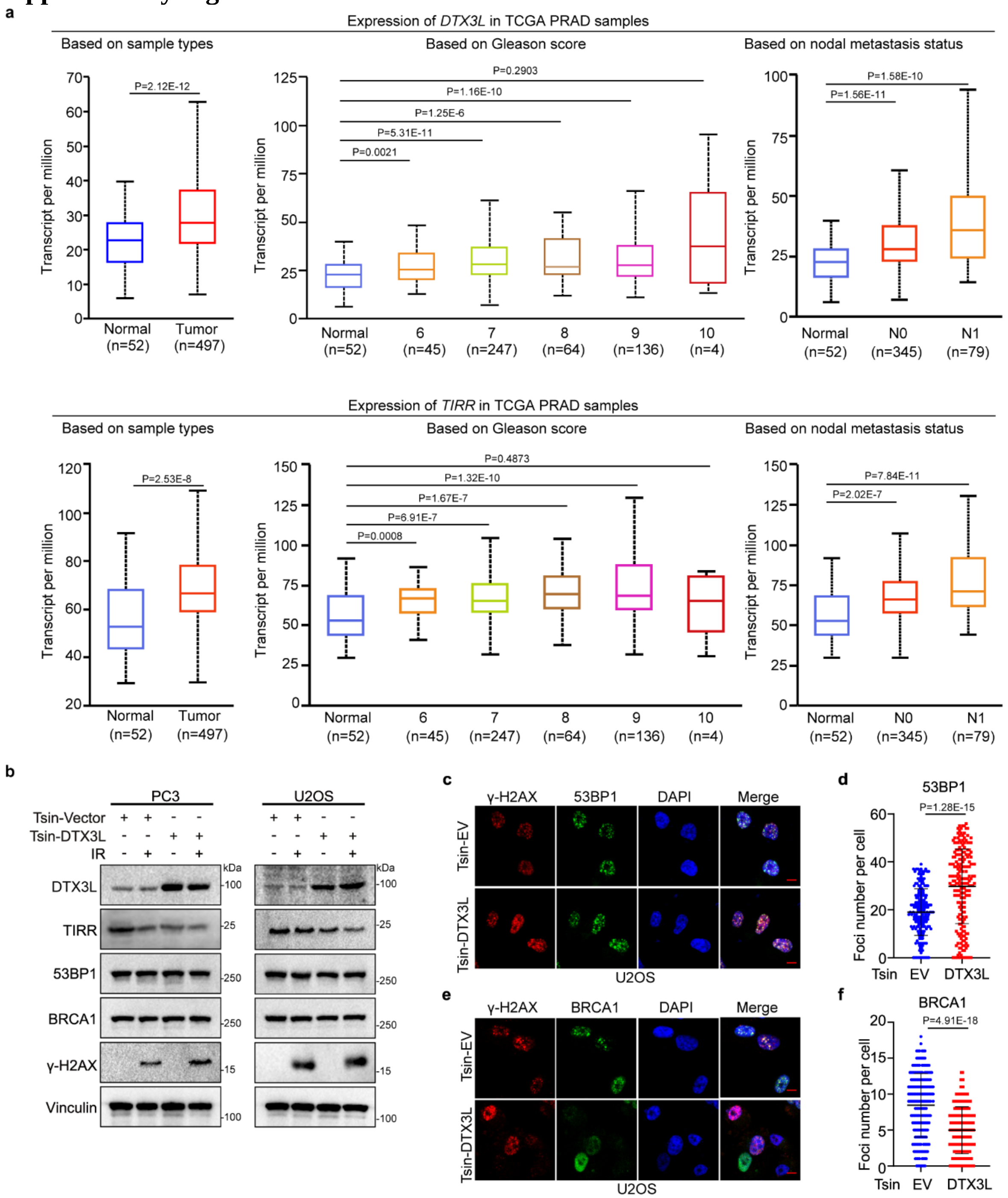

Supplementary Fig. 5 DTX3L overexpression impairs HR activity and promotes chromosomal instability in prostate cancer. Related to Fig. 6.

a Normalized expression of *DTX3L* or *TIRR* mRNA levels based on sample types, Gleason scores and metastasis status from TCGA prostate cancer dataset generated from UALCAN (https://ualcan.path.uab.edu/index.html). Means and SDs are represented. *P* values based on the order of appearance: 2.12E-12; 0.0021, 5.31E-11,1.25E-6, 1.16E-10, 0.2903; 1.56E-11, 1.58E-10; 2.53E-8; 0.0008; 6.91E-7, 1.67E-7, 1.32E-10, 0.4873; 2.02E-7, 7.84E-11.

b-f PC-3 and U2OS cells infected with lentivirus expressing EV or DTX3L were exposed to IR (8 Gy), followed by WB analysis of whole-cell lysates and IFC of 53BP1 (**c**) and BRCA1 (**e**). Scale bar, 10 μm. Similar results for (**b**) panel were obtained in three independent experiments. The average foci number (**d, f**) in each cell were quantified. Scale bar, 10 μm. Data were shown as the mean ± SD from three biological replicates (n = 200). Two-tailed unpaired Student's *t*-test; *P* values based on the order of appearance: 1.28E-15 (**d**); 4.91E-18(**f**). Source data are provided as a Source Data file.

Supplementary Figure 6

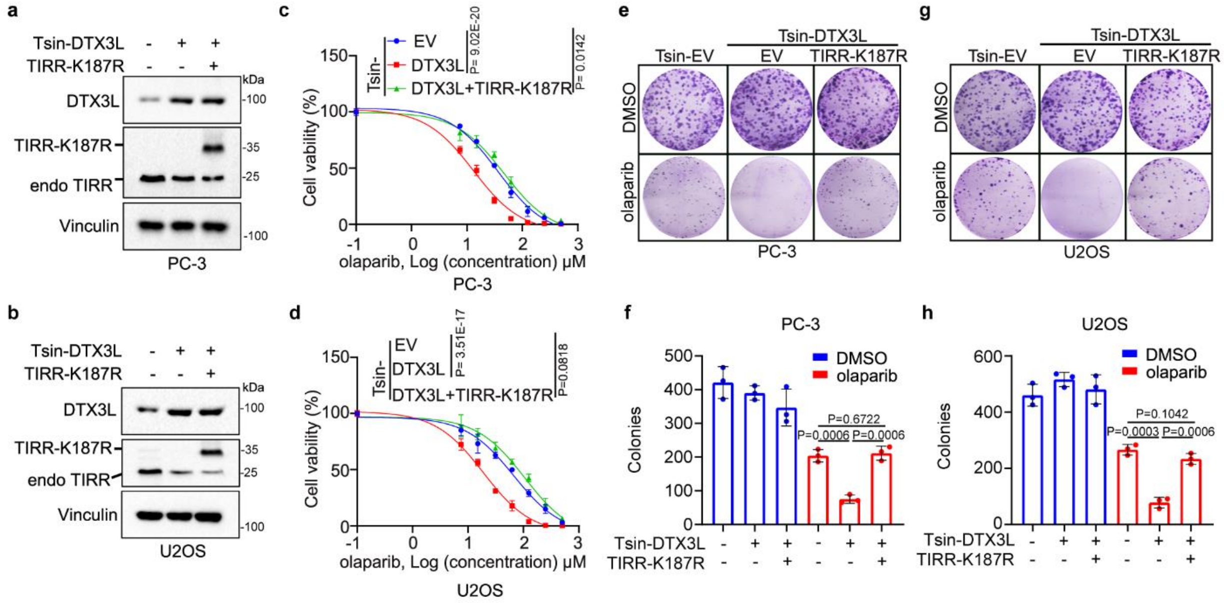

Supplementary Fig. 6 DTX3L expression sensitizes prostate cancer cells to synthetic lethality by PARP inhibitors. Related to Fig. 7.

a, b WB analysis of DTX3L and TIRR in whole-cell lysates from PC-3 (**a**) and U2OS (**b**) cells expressing either EV or DTX3L, and further transfected with EV or TIRR-K187R mutant. Similar results were obtained in three independent experiments.

c, d Dose-response survival curves of PC-3 (**c**) and U2OS (**d**) cells exposed to increasing concentrations of olaparib. Data were shown as the mean ± SD of three independent experiments (n = 3). Two-way ANOVA; *P* = 9.02E-20, F = 407.583, *P* = 0.0142, F = 6.727 (**c**); *P* = 3.51E-17, F = 271.129, *P* = 0.0818, F= 5.347 (**d**).

e-h Colony formation assays in PC-3 (**e, f**) and U2OS (**g, h**) cell lines treated with DMSO or olaparib. The number of colonies was counted. Representative colonies are shown in (**e**, **g**), with quantification data shown in (**f, h**). Data were presented as the mean ± SD of three independent experiments (n = 3). Two-tailed unpaired Student's *t*-test. *P* values based on the order of appearance: 0.0006, 0.6722, 0.0006 (**f**); 0.0003, 0.1042, 0.0006 (**h**). Source data are provided as a Source Data file.

Supplementary Table 1: Oligonucleotides. Related to Methods.

OLIGO NAME	SEQUENCE 5’-3’
TIRR-F	GAGCTGAAGCAGATCAGCCG
TIRR-R	CGTCGAAACGCATCTGCATC
18S-F	GGAGTATGGTTGCAAAGCTGA
18S-R	ATCTGTCAATCCTGTCCGTGT
FLAG-TIRR-M1-F	GCGGAGCAGGCGCACGCCGCGGAGGCCAGCGCGGTGCAC
FLAG-TIRR-M1-R	CGTCAGCTGCCGCGCG
FLAG-TIRR-M2-F	GCGGAGGTGGCGGGCCTCGCGCGGGCCCCGCTGTACACC
FLAG-TIRR-M2-R	GCCGTGGTCGCGCGAGT
FLAG-TIRR-M3-F	GCCCTCTTTGCCGCCAAGGTGGCCAACGCGATGCCCGAGGAG
FLAG-TIRR-M3-R	CTGGCACTTAGCCGTGCTCACG
HA-XPO1-I521A/L525A-F	GCAAAGGATCTAGCAGGATTATGTGAACAG
HA-XPO1-I521A/L525A-R	AACAGTAACAAGAAATCG
HA-XPO1-F561A-F	GCTCTGAAGACTGTAGTTAACAAGC
HA-XPO1-F561A-R	TTTCCAGTGAGCTCTC
HA-XPO1-F572A-F	GCCATGCATGAGACCCATGATG
HA-XPO1-F572A-R	TTCGAACAGCTTGTTAACTAC
HA-DTX3L-D1-F	ACTTCCTATGGTATTCAG
HA-DTX3L-D1-R	GATGCCCTTTTCCTTCTTGTC
HA-DTX3L-D2-F	TAAGCGGCCGCGGGGATC
HA-DTX3L-D2-R	CTGGCATGTGGGACAGATTG
HA-DTX3L-D3-F	TAAGCGGCCGCGGGGATC
HA-DTX3L-D3-R	CTTGTCCAGTTCTGAGGC
HA-DTX3L-C561S/C564S-F	AGTGTCATCAGTATGGACACCATTAGTAAC
HA-DTX3L-C561S/C564S-R	GATTGGCTTATATGACATGGC
DTX3L-PLVX3-ECORI-F	GAGGATCTATTTCCGGTGAATTATGGCCTCCCACCTGCGC
DTX3L-PLVX3-BAMHI-F	GGGAGAGGGGCGGATCTTACTCAATTCCTTTGGCTTTCAGCTCCT
