# Supplementary material for: DTX3L-mediated TIRR nuclear export and degradation regulates DNA repair pathway choice and PARP inhibitor sensitivity
Source: Nat Commun. 2024 Dec 5;15:10596. doi: 10.1038/s41467-024-54978-5 (PMC11618752; doi:10.1038/s41467-024-54978-5)
Supplement: Supplementary file 2 — Reporting Summary [file 41467_2024_54978_MOESM2_ESM.pdf]

nature portfolio

Corresponding author(s): Lei Li

Last updated by author(s): Oct 14, 2024

Reporting Summary

Nature Portfolio wishes to improve the reproducibility of the work that we publish. This form provides structure for consistency and transparency in reporting. For further information on Nature Portfolio policies, see our Editorial Policies and the Editorial Policy Checklist.

Statistics

For all statistical analyses, confirm that the following items are present in the figure legend, table legend, main text, or Methods section.

n/a	Confirmed	
☐	☒	The exact sample size (n) for each experimental group/condition, given as a discrete number and unit of measurement
☐	☒	A statement on whether measurements were taken from distinct samples or whether the same sample was measured repeatedly
☐	☒	The statistical test(s) used AND whether they are one- or two-sided<br>*Only common tests should be described solely by name; describe more complex techniques in the Methods section.*
☐	☒	A description of all covariates tested
☐	☒	A description of any assumptions or corrections, such as tests of normality and adjustment for multiple comparisons
☐	☒	A full description of the statistical parameters including central tendency (e.g. means) or other basic estimates (e.g. regression coefficient) AND variation (e.g. standard deviation) or associated estimates of uncertainty (e.g. confidence intervals)
☐	☒	For null hypothesis testing, the test statistic (e.g. F, t, r) with confidence intervals, effect sizes, degrees of freedom and P value noted<br>*Give P values as exact values whenever suitable.*
☒	☐	For Bayesian analysis, information on the choice of priors and Markov chain Monte Carlo settings
☐	☒	For hierarchical and complex designs, identification of the appropriate level for tests and full reporting of outcomes
☐	☒	Estimates of effect sizes (e.g. Cohen's d, Pearson's r), indicating how they were calculated

Our web collection on statistics for biologists contains articles on many of the points above.

Software and code

Policy information about availability of computer code

Data collection	Nikon Eclipse Ti-S Inverted Research Microscope and Olympus BX51 Microscope were used to take ICC pictures. CFX96 Touch™ System was used to perform quantitative PCR. Leica SCN400 was used to take IHC pictures. Microsoft Excel version 2010 was used to process the data. MicroCal iTC200 system was used to perform isothermal titration calorimetry (ITC) assay.
Data analysis	Graphing and statistical analysis: GraphPad Prism 8.0 (GraphPad, Inc.), ImageJ (version 1.53, NIH) or Microsoft Office Excel 2010. Mass spectrometry analysis were processed with the UniProt human protein database (75,004 entries, download on 07-01-2020) using Protein Discoverer (Version 2.4.1.15, Thermo Fisher Scientific) and Mascot (Version 2.7.0, Matrix Science). Isothermal titration calorimetry (ITC) analysis were processed with MicroCal PEAQ-ITC Analysis Software (version 1.41, Malvern Panalytical).

For manuscripts utilizing custom algorithms or software that are central to the research but not yet described in published literature, software must be made available to editors and reviewers. We strongly encourage code deposition in a community repository (e.g. GitHub). See the Nature Portfolio guidelines for submitting code & software for further information.

Data

Policy information about availability of data

All manuscripts must include a data availability statement. This statement should provide the following information, where applicable:

- Accession codes, unique identifiers, or web links for publicly available datasets
- A description of any restrictions on data availability
- For clinical datasets or third party data, please ensure that the statement adheres to our policy

All data needed to evaluate the conclusions in the paper are present in the paper and/or the Supplementary Materials. The raw mass spectrometry proteomics data have been deposited to the ProteomeXchange Consortium (http://proteomecentral.proteomexchange.org) via the iProX partner repository with the dataset identifier PXD052392 and PXD055432. Further information and requests for resources and reagents should be directed to and will be fulfilled by the lead contact, Lei Li (lilydr@163.com). Source data are provided with this paper.

Research involving human participants, their data, or biological material

Policy information about studies with human participants or human data. See also policy information about sex, gender (identity/presentation), and sexual orientation and race, ethnicity and racism.

Reporting on sex and gender	The prostate cancer (male-specific cancer type) tissue IHC slides were generated from only male patients, because the study investigated the pathology of prostate cancer.
Reporting on race, ethnicity, or other socially relevant groupings	All the experiments were designed without any biases on race, ethnicity, or other socially relevant groupings.
Population characteristics	Archive tumor tissue collected at the First Affiliated Hospital of Xi'an Jiaotong University (Xi'an, China). 44 cases (age from 50-80) were selected solely based on their histopathology diagnosis and tumor tissue availability.
Recruitment	Samples were collected retrospectively from the First Affiliated Hospital of Xi'an Jiaotong University and annotated for major clinicopathologic variables through review of pathology reports and clinical records by trained personnel. Archive tumor tissue were selected solely based on their histopathology diagnosis and tumor tissue availability, and no other biases were present.
Ethics oversight	The studies were approved by the Ethics Committee of the First Affiliated Hospital of Xi'an Jiaotong University (Xi'an, China).

Note that full information on the approval of the study protocol must also be provided in the manuscript.

Field-specific reporting

Please select the one below that is the best fit for your research. If you are not sure, read the appropriate sections before making your selection.

☒ Life sciences ☐ Behavioural & social sciences ☐ Ecological, evolutionary & environmental sciences

For a reference copy of the document with all sections, see nature.com/documents/nr-reporting-summary-flat.pdf

Life sciences study design

All studies must disclose on these points even when the disclosure is negative.

Sample size	No sample size calculation was performed. Sample size was determined from similar experiments in the literature and for all experiments a minimum of three technical replicates were analyzed per sample.
Data exclusions	No data were excluded from the analyses.
Replication	Data are presented as the mean ± SD. Replicates of experiments are specified in figure legends.
Randomization	In mouse experiments, all samples and animals are randomly divided into different experimental groups as indicated. For other studies, experiments were performed in large cell line populations, and randomization was therefore not appropriate, but the treated groups were attributed randomly.
Blinding	For mouse study, investigators were blinded to group allocation during data collection and analysis. For other studies, experiments were performed blinded.

Reporting for specific materials, systems and methods

We require information from authors about some types of materials, experimental systems and methods used in many studies. Here, indicate whether each material, system or method listed is relevant to your study. If you are not sure if a list item applies to your research, read the appropriate section before selecting a response.

Materials & experimental systems

n/a	Involved in the study
	☒ Antibodies
	☒ Eukaryotic cell lines
☒	Palaeontology and archaeology
	☒ Animals and other organisms
☒	Clinical data
☒	Dual use research of concern
☒	Plants

Methods

n/a	Involved in the study
☒	ChIP-seq
☒	Flow cytometry
☒	MRI-based neuroimaging

Antibodies

Antibodies used	Primary antibodies used include NUDT16L1 (Sigma-Aldrich, # HPA044186, 1:1000), 53BP1 (Abcam, # ab36823, 1:1000), 53BP1 (Santa Cruz, # sc-515841, 1:500), XPO1 (Cell Signaling, # 46249,1:1000), Vinculin (Santa Cruz, # sc-73614, 1:1000), Lamin B1 (ABclonal, # A16909, 1:1000), β-tublin (ABclonal, # AC021, 1:1000), DTX3L (Santa Cruz, # sc-514776, 1:1000), Myc (Santa Cruz, # sc-40, 1:1000), Flag (Cell Signaling, # 8146, 1:1000), Flag-Alexa Fluor 594 (MBL, # M185-A59, 1:1000), HA (Cell Signaling, # 3724, 1:1000), Ubiquitin (Cell Signaling, # 14049, 1:1000), Phospho histone H2A.X (S139) (Cell Signaling, # 9718, 1:1000), and Phospho histone H2A.X (S139) (Cell Signaling, # 80312S, 1:1000). Second antibodies were Rabbit IgG (H+L), FITC (Xi'an Zhuangzhi Biotechnology Co., Ltd., # EK023, 1:500), Rabbit IgG (H+L), Cy3 (Xi'an Zhuangzhi Biotechnology Co., Ltd., # EK022, 1:500), Mouse IgG (H+L), FITC (Xi'an Zhuangzhi Biotechnology Co., Ltd., # EK013, 1:500), Mouse IgG (H+L), Cy3 (Xi'an Zhuangzhi Biotechnology Co., Ltd., # EK012, 1:500), Rabbit IgG (Abclonal, # AS014, 1:5000), and Mouse IgG (Abclonal, # AS003, 1:5000).
Validation	NUDT16L1 (Sigma-Aldrich, # HPA044186, 1:1000), https://www.sigmaaldrich.cn/CN/en/product/sigma/hpa044186;<br>53BP1 (Abcam, # ab36823, 1:1000), https://www.abcam.com/products/primary-antibodies/53bp1-antibody-ab36823.html;<br>53BP1 (Santa Cruz, # sc-515841, 1:500), https://www.scbt.com/p/53bp1-antibody-e-10;<br>XPO1 (Cell Signaling, # 46249,1:1000), https://www.cellsignal.com/products/primary-antibodies/exportin-1-crm1-d6v7n-rabbit-mab/46249;<br>Vinculin (Santa Cruz, # sc-73614, 1:1000), https://www.scbt.com/p/vinculin-antibody-7f9?requestFrom=search;<br>Lamin B1 (ABclonal, # A16909, 1:1000), https://abclonal.com.cn/catalog/A1910;<br>β-tublin (ABclonal, # AC021, 1:1000), https://abclonal.com.cn/catalog/AC021;<br>DTX3L (Santa Cruz, # sc-514776, 1:1000), https://www.scbt.com/p/dtx3l-antibody-d-10;<br>Myc (Santa Cruz, # sc-40, 1:1000), https://www.scbt.com/p/c-myc-antibody-9e10;<br>Flag (Cell Signaling, # 8146, 1:1000), https://www.cellsignal.com/products/primary-antibodies/dykddddk-tag-9a3-mouse-mab-binds-to-same-epitope-as-sigma-s-anti-flag-m2-antibody/8146;<br>Flag-Alexa Fluor 594 (MBL, # M185-A59, 1:1000), https://www.mblbio.com/bio/g/dtl/A/index.html?pcd=M185-A59;<br>HA (Cell Signaling, # 3724, 1:1000), https://www.cellsignal.cn/products/primary-antibodies/ha-tag-c29f4-rabbit-mab/3724;<br>Ubiquitin (Cell Signaling, # 14049, 1:1000), https://www.cellsignal.cn/products/antibody-conjugates/ubiquitin-p4d1-mouse-mab-hrp-conjugate/14049;<br>Phospho histone H2A.X (S139) (Cell Signaling, # 9718, 1:1000), https://www.cellsignal.com/products/primary-antibodies/phosphohistone-h2a-x-ser139-20e3-rabbit-mab/9718;<br>Phospho histone H2A.X (S139) (Cell Signaling, # 80312S, 1:1000), https://www.cellsignal.com/products/primary-antibodies/phosphohistone-h2a-x-ser139-d7t2v-mouse-mab/80312;<br>Rabbit IgG (H+L), FITC (Xi'an Zhuangzhi Biotechnology Co., Ltd., # EK023, 1:500), http://www.zhuangzhibio.com/index.php?id=1026;<br>Rabbit IgG (H+L), Cy3 (Xi'an Zhuangzhi Biotechnology Co., Ltd., # EK022, 1:500), http://www.zhuangzhibio.com/index.php?id=110;<br>Mouse IgG (H+L), FITC (Xi'an Zhuangzhi Biotechnology Co., Ltd., # EK013, 1:500), http://www.zhuangzhibio.com/index.php?id=1025;<br>Mouse IgG (H+L), Cy3 (Xi'an Zhuangzhi Biotechnology Co., Ltd., # EK012, 1:500), http://www.zhuangzhibio.com/index.php?id=112;<br>Rabbit IgG (Abclonal, # AS014, 1:5000), https://abclonal.com.cn/catalog/AS014;<br>Mouse IgG (Abclonal, # AS003, 1:5000), https://abclonal.com.cn/catalog/AS003;<br>All of the antibodies used in this study were validated for the use in human specimens by the manufacturers and for the respective methods used in this manuscript (see home pages of respective manufacturers using catalogue numbers provided above).

Eukaryotic cell lines

Policy information about cell lines and Sex and Gender in Research

Cell line source(s)	The immortalized human embryonic kidney cell line 293T (CRL-11268) and prostate cancer cell lines PC-3 (CRL-1435) and human osteosarcoma cell line U2OS (HTB-96) were purchased from ATCC (Manassas, VA).
Authentication	The cell lines were authenticated periodically via STR profiling (IDEXX BioResearch).
Mycoplasma contamination	All cell lines were tested negative of mycoplasma contamination.
Commonly misidentified lines (See ICLAC register)	None commonly misidentified cell lines were used.

Animals and other research organisms

Policy information about studies involving animals; ARRIVE guidelines recommended for reporting animal research, and Sex and Gender in Research

Laboratory animals	6 weeks old NSG male mice were used for prostate cancer xenograft study as described in the Methods section. Mice were housed in standard cages with an SPF environment with a 12-hour light/dark cycle at a room temperature of 22°C±2°C, humidity of 50%±5%, with free access to food and water.
Wild animals	No
Reporting on sex	Only male mice were used in this study, because the study investigated the potential therapeutic use of PARP inhibitors in prostate cancer (male specific cancer type).
Field-collected samples	No
Ethics oversight	The animal study was approved by the Institutional Animal Care and Use Committee (IACUC) at the First Affiliated Hospital of Xi'an Jiaotong University (Xi'an, China).

Note that full information on the approval of the study protocol must also be provided in the manuscript.

Plants

Seed stocks	N/A
Novel plant genotypes	N/A
Authentication	N/A
